# Arterial Effects of Canakinumab in Patients With Atherosclerosis and Type 2 Diabetes or Glucose Intolerance

**DOI:** 10.1016/j.jacc.2016.07.768

**Published:** 2016-10-18

**Authors:** Robin P. Choudhury, Jacqueline S. Birks, Venkatesh Mani, Luca Biasiolli, Matthew D. Robson, Philippe L. L’Allier, Marc-Alexandre Gingras, Nadia Alie, Mary Ann McLaughlin, Craig T. Basson, Alison D. Schecter, Eric C. Svensson, Yiming Zhang, Denise Yates, Jean-Claude Tardif, Zahi A. Fayad

**Affiliations:** a Oxford Acute Vascular Imaging Centre, Radcliffe Department of Medicine, University of Oxford, Oxford, United Kingdom; b Centre for Statistics in Medicine, Nuffield Department of Orthopaedics, Rheumatology and Musculoskeletal Sciences, University of Oxford, Botnar Research Centre, Oxford, United Kingdom; c Translational and Molecular Imaging Institute, Icahn School of Medicine at Mount Sinai, New York, New York; d Montreal Heart Institute, Université de Montréal, Montreal, Canada; e Department of Medicine, Université de Montréal, Montreal, Canada; f Novartis Institutes for BioMedical Research, Cambridge, Massachusetts

**Keywords:** C-reactive protein, homeostasis model assessment, inflammation, interleukin-1, CI, confidence interval, GMR, geometric mean ratio, HOMA, homeostasis model assessment, HbA_1c_, glycosylated hemoglobin, hs-CRP, high-sensitivity C-reactive protein, IGT, impaired glucose tolerance, IL, interleukin, IL-1RA, interleukin-1 receptor antagonist, MRI, magnetic resonance imaging, PWV, pulsed wave velocity, T2DM, type 2 diabetes mellitus

## Abstract

**Background:**

Evidence suggests that interleukin (IL)-1β is important in the pathogenesis of atherosclerosis and its complications and that inhibiting IL-1β may favorably affect vascular disease progression.

**Objectives:**

The goal of this study was to evaluate the effects of IL-1β inhibition with canakinumab versus placebo on arterial structure and function, determined by magnetic resonance imaging.

**Methods:**

Patients (N = 189) with atherosclerotic disease and either type 2 diabetes mellitus or impaired glucose tolerance were randomized to receive placebo (n = 94) or canakinumab 150 mg monthly (n = 95) for 12 months. They underwent magnetic resonance imaging of the carotid arteries and aorta.

**Results:**

There were no statistically significant differences between canakinumab compared with placebo in the primary efficacy and safety endpoints. There was no statistically significant change in mean carotid wall area and no effect on aortic distensibility, measured at 3 separate anatomic sites. The change in mean carotid artery wall area was –3.37 mm^2^ after 12 months with canakinumab versus placebo. High-sensitivity C-reactive protein was significantly reduced by canakinumab compared with placebo at 3 months (geometric mean ratio [GMR]: 0.568; 95% confidence interval [CI]: 0.436 to 0.740; p < 0.0001) and 12 months (GMR: 0.56; 95% CI: 0.414 to 0.758; p = 0.0002). Lipoprotein(a) levels were reduced by canakinumab compared with placebo (–4.30 mg/dl [range: –8.5 to –0.55 mg/dl]; p = 0.025] at 12 months), but triglyceride levels increased (GMR: 1.20; 95% CI: 1.046 to 1.380; p = 0.01). In these patients with type 2 diabetes mellitus or impaired glucose tolerance, canakinumab had no effect compared with placebo on any of the measures assessed by using a standard oral glucose tolerance test.

**Conclusions:**

There were no statistically significant effects of canakinumab on measures of vascular structure or function. Canakinumab reduced markers of inflammation (high-sensitivity C-reactive protein and interleukin-6), and there were modest increases in levels of total cholesterol and triglycerides. (Safety & Effectiveness on Vascular Structure and Function of ACZ885 in Atherosclerosis and Either T2DM or IGT Patients; NCT00995930)

Atherosclerosis is well-established as a disease with an important inflammatory component 1, 2, 3. Systemic markers of inflammation such as C-reactive protein and serum amyloid A are strongly related to cardiovascular prognosis in various populations and clinical settings 4, 5. Furthermore, therapeutic interventions that reduce cardiovascular risk have also been associated with a reduction in systemic inflammatory markers 6, 7. However, whether specifically targeting inflammation reduces cardiovascular risk remains unknown.

Interleukins are important mediators of inflammation, both locally and systemically. Macrophages are key cellular components of atherosclerotic plaque and produce interleukin (IL)-1β (8), which is also promoted by cellular cholesterol activation of inflammasomes (9). IL-1β and interleukin-1α exert proinflammatory effects that are inhibited by the endogenous antagonist interleukin-1 receptor antagonist (IL-1RA). Atherosclerosis-prone mice that are deficient in IL-1β develop smaller lesions (10), and administration of IL-1RA reduces early atherogenesis in mice (11). IL-1RA–deficient mice have shown increased atherosclerosis (12) and vascular inflammation, associated with destruction of elastic tissues (13).

Therefore, given the key role for IL-1β as a mediator of innate immunity and the effects of interleukin inhibition in experimental atherosclerosis, interventions to reduce inflammation through IL-1β have been proposed to treat atherosclerosis. Although evidence of benefit to vascular disease in humans remain sparse, administration of the IL-1RA anakinra to patients with rheumatoid arthritis improved several measures of vascular function, including aortic distensibility, flow-mediated vasodilation, and coronary flow reserve (14).

In addition to its key role in vascular disease, IL-1β has been implicated in the pathogenesis of type 2 diabetes mellitus (T2DM). IL-1RA expression is reduced in pancreatic islets of patients with T2DM, and high glucose concentrations induce the production of IL-1β in human pancreatic beta cells, leading to impaired insulin secretion, decreased cell proliferation, and apoptosis (15). Blockade of the interleukin-1 receptor with anakinra improved glycemia and beta-cell secretory function and reduced markers of systemic inflammation in patients with T2DM (16). Patients with T2DM are at high risk for cardiovascular disease (17) and have evidence of both increased plaque inflammation (18) and reduced arterial distensibility (19). This group might, therefore, derive “metabolic” and “vascular” benefits from targeting IL-1β, including reduced risk of atherothrombotic complications.

A human monoclonal anti-human IL-1β antibody of the immunoglobulin G1/k isotype canakinumab functionally neutralizes IL-1β through steric hindrance of its receptor interaction. It is effective in reducing systemic markers of inflammation, including C-reactive protein and IL-6 (20). Its effects on cardiovascular outcomes are under investigation in the CANTOS (Canakinumab Anti-inflammatory Thrombosis Outcomes Study) trial (21).

Vascular magnetic resonance imaging (MRI) has emerged as a precise, highly reproducible, and versatile tool to assess both vascular structure and function at multiple arterial loci 22, 23, 24, 25. Accordingly, we designed a randomized, placebo-controlled Phase II clinical trial to test the effects of IL-1β inhibition, using canakinumab, on: 1) MRI-derived measures of vascular structure and function; 2) measures of diabetes control; and 3) indicators of systemic inflammation in patients with atherosclerotic vascular disease and impaired glucose tolerance (IGT) or T2DM.

## Methods

Novartis Pharmaceuticals (Cambridge, Massachusetts) initiated this Phase II, double-blind, randomized, placebo-controlled trial, with the final study protocol designed in collaboration with the investigators (R.P.C., J.-C.T., and Z.A.F.), based on their previously published methods 19, 23, 24. The study was undertaken at 9 centers in Canada, the United Kingdom, the United States, Germany, and Israel, in compliance with the principles of the Declaration of Helsinki and according to Good Clinical Practice guidelines. The protocol was reviewed and approved by the institutional review board, or equivalent, for each center. All participants provided written informed consent before undertaking any study procedures.

### Patients

Patients (ages 18 to 74 years) were eligible for inclusion if they had clinically evident atherosclerotic vascular disease: previous myocardial infarction; history of angina; carotid stenosis (>30%); peripheral vascular disease (ankle–brachial index <0.9); endarterectomy >3 months previously; or transient ischemic attack or stroke. In addition, patients must also have had either T2DM (for ≤14 years and glycosylated hemoglobin [HbA_1c_] levels between 6% and 10%) or IGT (defined as a peak 2-h glucose reading ≥140 mg/dl but <200 mg/dl after an oral glucose tolerance test during screening). Patients were to have been on stable statin therapy for a period of ≥6 weeks before screening (or have physician-documented statin intolerance).

After consent, eligible patients were randomized (1:1) to receive canakinumab 150 mg or placebo, subcutaneously, monthly for 12 months. Exclusion criteria included: pregnancy; systemic steroid use; baseline high-sensitivity C-reactive protein (hs-CRP) levels >30 mg/l; history of significant multiple drug allergies; history or evidence of chronic infection, including tuberculosis and liver disease; or a standard contraindication to MRI. Randomization occurred between December 2009 and November 2012. Patient groups were assigned centrally according to a validated computer-generated randomization code, stratified according to glycemic status (T2DM or IGT).

### Imaging procedures

An integrated vascular MRI was performed at baseline and after 3 and 12 months of treatment. If the imaging data did not meet an evaluable standard at any time point, the patient was rescanned. Randomization required an evaluable baseline scan. The imaging procedure comprised measures of aortic wall area and distensibility, as well as carotid wall area bilaterally. The imaging protocol was adapted from Lee et al. (19), and staff at each imaging site underwent individualized training to ensure consistency of method and data acquisition. All trial sites used a 3.0-T whole-body MRI scanner, including Trio, TIM Trio, or Verio (Siemens Medical Solutions USA, Inc., Malvern, Pennsylvania) or Achieva (Philips, Amsterdam, the Netherlands) platforms. For carotid imaging, a bilateral 4-channel carotid array (Machnet B.V., Roden, the Netherlands) was used on the Siemens scanners and an equivalent multi-channel (4 to 8) phased array carotid coil (Shanghai Medical, Shanghai, China) was used on the Philips scanners.

Detailed information regarding imaging protocols and analysis is available in the Online Appendix.

The velocity at which the arterial pulse propagates is termed pulsed wave velocity (PWV). A measure of arterial stiffness, it is an independent predictor of mortality in both T2DM and IGT (26). The Sphygmocor platform (AtCor Medical Pty. Ltd., West Ryde, Australia) was applied immediately before MRI scanning to acquire the aortic central pulse pressure from radial artery applanation tonometry and the PWV from the carotid-femoral pulse waves.

### Safety assessments

Monitoring of vital signs, electrocardiogram, standard hematology, and biochemistry (including measurement of lipoproteins, liver function, and creatine kinase) were conducted throughout the study. A data monitoring committee oversaw subject safety on an ongoing basis. In addition, 3 adjudication committees made blinded assessments of adverse events in relation to cardiac, malignant, and infection-related events.

### Endpoints

The primary efficacy objectives were the effects of the drug on aortic distensibility and total plaque burden in the aorta and carotid arteries; the primary safety objective was the safety and tolerability of canakinumab in this population. Secondary objectives included the effects of canakinumab on aortic PWV; hs-CRP; HbA_1c;_ homeostasis model assessment (HOMA)–insulin resistance; and peak blood glucose level 2 h after an oral glucose challenge. Exploratory analyses of peripheral biomarkers of inflammation (including IL-6, serum amyloid A, and plasma lipoproteins) were also performed.

### Statistical analyses

Sample size was calculated from the study of Lee et al. (19). To detect a 35% change in aortic distensibility or an 11% change in plaque burden at 12 months to achieve a power of 0.8 and nominal p < 0.05 (2-sided), 60 patients per group were required. To ensure 120 datasets with 12-month follow-up data, it was planned to randomize 190 patients. Given the exploratory nature of the study, corrections were not made for the multiplicity of statistical tests. As defined by the protocol, patients were included in the 3-month data analysis if they had no missing doses at 3 months; for the 12-month analysis, participants were required to have no missing doses by 3 months and 1 or no missing doses between 3 and 12 months.

To compare treatment with placebo, we conducted an analysis of covariance on change from baseline, including the glycemic status as a factor and the baseline as a covariate at 3 and 12 months. All data were checked for normality and log-transformed, if appropriate. When a log transformation of the change from baseline was not possible because of negative values, an analysis of covariance was conducted on the log-transformed 3- and 12-month data, including glycemic index status as a factor and the log-transformed baseline as a covariate. We considered a p value <0.05 as significant. Results are reported as means with 95% confidence limits. Two interim analyses were pre-specified, when n = 60 and all patients had completed 3 months of treatment, respectively, with the intention of halting the study if adverse measures were identified or for futility but not for interim positive efficacy. The interim analyses were performed by independent personnel not directly associated with the study’s conduct.

The study sponsor and funder (Novartis) participated in discussions about the design and conduct of this study; they also provided the drugs used in the trial and logistical support for its execution. The trial design, endpoints, and statistical analyses were largely derived from the academic investigators’ previously published studies 19, 23, 24. Following the final database lock, all patient data were analyzed independently by the Centre for Statistics in Medicine, Oxford (J.B.). The manuscript was drafted by the academic investigators (R.P.C., J.S.B., J.-C.T., and Z.A.F.), in accordance with the written agreement between Novartis and the academic institutions, and reviewed and revised by the writing committee. All authors had full access to all the data in the study and assume responsibility for publication.

All statistical analyses were performed by using Stata 14 (StataCorp LP, College Station, Texas).

## Results

Of 450 patients screened, 189 were randomized to receive either placebo (n = 94) or canakinumab 150 mg (n = 95). The proportions of patients with diabetes, duration of diabetes, and glycemic control (estimated from HbA1_c_) were similar in the groups, and there was a high prevalence for each of hypertension, dyslipidemia, and background coronary artery disease (Table 1); the latter was slightly higher proportionately in the placebo group. There was no significant difference between the canakinumab group compared with the placebo group for the overall rate of completion of the study, which was 70.5% with canakinumab versus 77.7% for placebo (risk ratio: 1.32; 95% confidence interval [CI]: 0.81 to 2.15; p = 0.26) or the rate of discontinuation due to adverse events (14.7% vs. 11.7%; risk ratio: 1.26; 95% CI: 0.60 to 2.63; p = 0.54) (Table 2). The most common cause for discontinuation was for adverse events. A full list of adverse events is provided in Online Table 1. Seven patients withdrew consent, and 7 patients were excluded from analysis due to significant protocol deviation.

**Figure 1:**
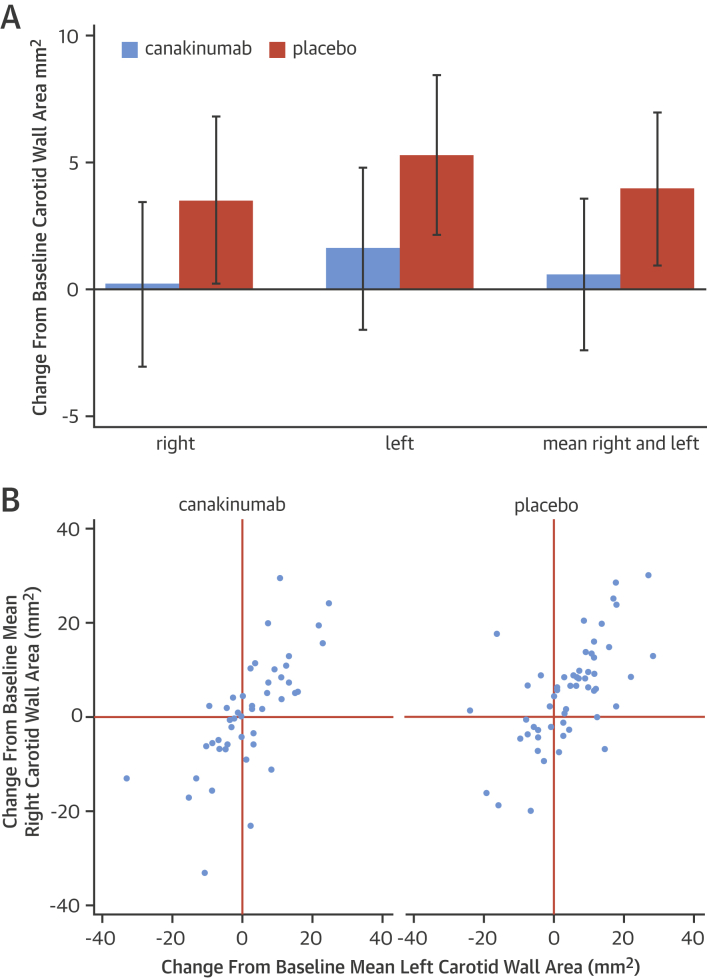
Changes in Carotid Wall Area We evaluated 12-month change from baseline in wall area as an indicator of atherosclerosis progression. **(A)** In both left and right carotid arteries, canakinumab retarded progression of wall area compared with placebo. Consistent in magnitude and direction, the changes did not reach statistical significance in the mean carotid wall area (pre-stated endpoint) or in either carotid artery analyzed separately. **Bars** = means with 95% confidence intervals. **(B)** Within the same patient, there was a striking concordance of change in wall area between left- and right-sided arteries, which was maintained in both treatment groups (p < 0.0001 for each). **Upper right quadrant** = patients with progression in both carotid arteries.

As a biomarker of atherosclerotic plaque burden, vessel wall area was quantified in the aorta and carotid arteries (Table 3). There was no statistically significant difference in mean carotid wall area between these 2 groups at either time point. Baseline mean carotid wall areas were 27.7 ± 9.79 mm^2^ and 27.1 ± 9.6 mm^2^ (p = NS) for the canakinumab and placebo groups, respectively. Change in mean carotid artery wall area was –3.37 mm^2^ (p = 0.06) after 12 months for canakinumab versus placebo. There was an increase (12 months vs. baseline) in wall area for each of the left and right carotid arteries (and mean of the left and right) with placebo but, on average, neither progression nor regression in the canakinumab group (Figure 1A). Change from baseline at 12 months was compared for individual patients between the left and right carotid arteries for both treatment groups. In each, there were strong correlations between changes in the left and right carotid arteries for canakinumab and placebo, respectively (p < 0.0001 for both) (Figure 1B). The vessel lumen area was not changed by canakinumab treatment. There was no statistically significant difference in wall area between canakinumab treatment and placebo at any of the 3 aortic sites at either 3 or 12 months.

Aortic distensibility was calculated from measurements made at 3 sites in the aorta. There were no statistically significant differences between canakinumab treatment and placebo for change in aortic distensibility, and no significant changes occurred in systolic or diastolic blood pressure at either 3 or 12 months of treatment versus baseline. There were also no significant differences in measures of PWV between these 2 groups at either time point (Table 3).

**Figure 2:**
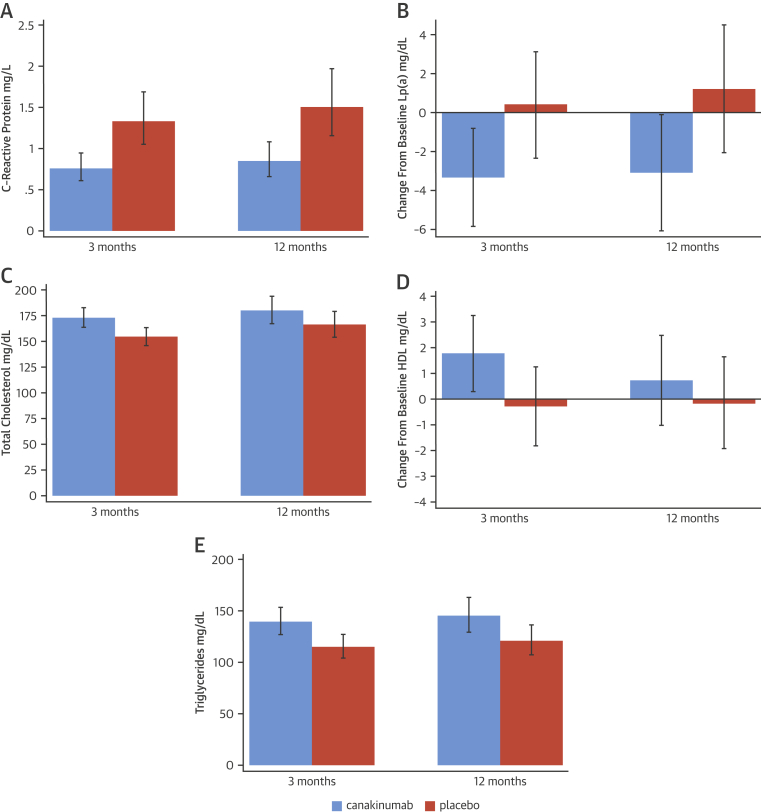
Changes in Lipids and C-Reactive Protein Absolute level or change from baseline at 3 and 12 months are shown for **(A)** C-reactive protein; **(B)** lipoprotein(a) (Lp[a]); **(C)** total cholesterol; **(D)** high-density lipoprotein (HDL) cholesterol; and **(E)** triglycerides.

Compared with placebo, canakinumab reduced hs-CRP at 3 months (geometric mean ratio [GMR]: 0.568; 95% CI: 0.436 to 0.740; p < 0.0001) and 12 months (GMR: 0.56; 95% CI: 0.414 to 0.758; p = 0.0002) (Figure 2). Similarly, IL-6 was reduced by canakinumab at the 3-month time point (GMR: 0.580; 95% CI: 0.483 to 0.697; p < 0.0001). Neither serum amyloid A nor adiponectin changed significantly in response to canakinumab treatment compared with placebo (Table 4).

In this study population of patients with T2DM or IGT and near-universal statin use, canakinumab had no effect on either plasma low-density lipoprotein or high-density lipoprotein cholesterol levels compared with placebo at either of the 3- or 12-month time points. Levels of total plasma cholesterol were mildly elevated from baseline in the canakinumab-treated patients at 3 months compared with the placebo group (GMR: 1.120; 95% CI: 1.050 to 1.195; p = 0.0008) but not at 12 months (GMR: 1.084; 95% CI: 0.991 to 1.185; p = 0.08). The elevation in total cholesterol level most likely reflected an elevation in triglyceride-rich lipoproteins, given the coinciding elevation in triglyceride level that also accompanied canakinumab treatment compared with placebo at 3 months (GMR: 1.21; 95% CI: 1.082 to 1.358; p = 0.001) and 12 months (GMR: 1.20; 95% CI: 1.046 to 1.380; p = 0.01).

Lipoprotein(a) levels were reduced by canakinumab compared with placebo. Changes from baseline values were as follows: at 3 months, mean change was –3.719 mg/dl (95% CI: –6.809 to –0.628; p = 0.02); at 12 months, mean change was –4.300 mg/dl (95% CI: –8.052 to –0.548; p = 0.025).

In this population of patients with T2DM (86%) or IGT (14%) and with median baseline HbA_1c_ levels <7%, canakinumab also had no significant effect compared with placebo on fasting blood glucose, HbA_1c_, HOMA–insulin resistance or HOMA-β, or 2-h glucose, obtained as part of an oral glucose tolerance test.

Major adverse cardiovascular events occurred in 9.0% of patients, with no significant difference between the active treatment group (11%) compared with the placebo group (7%) (risk ratio: 1.41; 95% CI: 0.56 to 3.56; p = 0.46) (Table 5). A full list of adverse events is given in Online Table 1.

## Discussion

Inflammation contributes to the pathogenesis of vascular dysfunction and atherogenesis, as well as to the complications of atherosclerosis (1). Data from preclinical studies have suggested that inhibiting IL-1β may directly affect atherosclerosis and vascular inflammation (10).

In the present randomized clinical trial of IL-1β inhibition in patients with T2DM or IGT, canakinumab 150 mg monthly reduced blood levels of IL-6 and hs-CRP (Central Illustration). Similar reductions in inflammatory indexes have been reported previously in patients with T2DM (20). Significantly, given the target population, the present study showed that this effect persists even with near-universal statin use. Canakinumab had no effect on fasting glucose, HbA_1c_, or measures of insulin sensitivity.

In common with earlier studies 19, 23, 24, atherosclerosis burden was quantified in the common carotid arteries and the aorta. We found no statistically significant effect of 12 months’ treatment with canakinumab on magnetic resonance–derived measures of vascular structure or function. However, in each of the common carotid arteries individually, and in the combined vessel average, there was a suggestion of possible retarded progression of atherosclerotic burden.

Because atherosclerosis is a systemic disease, we performed a further analysis, comparing changes in the right and left carotid arteries within individual patients. There were strong relationships between changes in the left- versus right-sided vessels evident in both the canakinumab and the placebo groups but with a tendency toward progression bilaterally in the placebo group. As detailed earlier, analyses of wall area were conducted by operators who were blinded to the timing and treatment allocation of the images. Moreover, measurements were made separately on individual slices, but the data presented are for whole arteries. Given this high level of analytical stringency, these consistent correlations supported the technical robustness of the measurements.

In common with previous studies, we chose to quantify vessel wall area in the tubular carotid arteries because they are less susceptible to error in serial measurements due to “volume averaging” effects. Previously described techniques allow plaque lipid quantification with the use of T2 mapping, but these were not current at the time of protocol design (27). Lipid elements may be the most readily mobilized components of atherosclerotic plaque, although the mechanisms by which IL-1β inhibition might affect plaque lipid are not clear. Vascular ^18^fludeoxyglucose positron emission tomography has been used effectively to evaluate carotid and aortic plaque macrophage activity in clinical trials, and this test may have been an alternative imaging modality 23, 28, 29. However, changes in macrophage function are associated with changes in substrate utilization and mode of energy generation (30). Therefore, using ^18^fludeoxyglucose positron emission tomography to identify macrophages on the basis of their glycolytic activity may provide only partial insight into the relevant biology with respect to IL-1β inhibition, and could even be misleading (31).

This study focused on changes in vessel wall structure and function. In doing so, we intended to obtain insights into the possible effects of canakinumab on different manifestations, or stages, of vessel wall pathology. Despite this expansive approach, we observed no significant effects on the vessel wall. Given the discordance with animal studies, one should also consider the possibility that previously observed beneficial effects of IL-1β inhibition need not have been realized directly at the arterial level. Accumulating evidence suggests roles for peripheral monocytes in accelerating atherosclerosis in response to inflammatory stimuli (32). Indeed, IL-1β enhances hematopoietic stem cell proliferation and leukocyte production after acute myocardial infarction in mice and is reduced by administration of anti–IL-1β antibodies (33).

In mice, most studies of IL-1β inhibition have shown reduced atherosclerosis and/or plaque inflammation; however, 1 study found that inactivation of IL-1 signaling through loss of the IL-1 receptor type 1 in apolipoprotein E^–/–^ mice promoted multiple indexes of atherosclerotic plaque instability, including reduced plaque smooth muscle cell content, reduced plaque collagen content, and impaired outward vessel remodeling, leading to reduced lumen size (34). Our study in humans found no evidence of changes in aortic characteristics according to distensibility measures at multiple sites. In theory, it would be possible for changes in lumen area/outward remodeling to be present despite no change in wall area, because wall area is derived by subtracting the lumen area from the total vessel area. Examining these measures individually provides information on tendencies for luminal constriction or outward remodeling. In human atherosclerosis, we found no change in lumen area or adverse effect on outward vessel remodeling.

Canakinumab had no effect on fasting glucose, HbA_1c_, or measures of insulin sensitivity in the present study. This outcome does not agree with the findings of Larsen et al. (16), who used the IL-1RA anakinra. The apparent discrepancy may reflect better glycemic control at baseline in our study, in which >50% of patients had HbA_1c_ levels <7% compared with a mean HbA_1c_ level >8.5% in the treatment arm of the anakinra trial. However, canakinumab administration did result in an increase in plasma triglyceride levels, as reported previously for this agent (20). The underlying mechanism is not clear. Other treatments targeting inflammation have also been associated with elevated levels of very-low-density lipoprotein triglycerides (35). There was also a marginal but statistically significant elevation in total cholesterol levels, with an increase by 18.5 mg/dl after 3 months’ treatment. Conversely, lipoprotein(a) was significantly reduced by canakinumab at both time points.

### Study limitations

This study did not evaluate atherosclerosis at the carotid bifurcation, where the burden of disease is often greatest, nor did it quantify plaque lipid content. Although MRI scans provide highly reproducible measures of plaque structure and vessel function, they offer no direct measure of plaque inflammation. It is possible for drugs to markedly affect plaque biology without altering plaque size, particularly on short-term follow-up (36). In this study, no attempt was made to select subjects on the basis of inflammatory status. More refined patient selection may enhance the effectiveness of drugs that are directed toward specific processes and pathways. Finally, we cannot exclude the possibility that the dose of canakinumab was not high enough to generate a maximal effect on atherosclerotic burden, although the dose was sufficient to lower both hs-CRP and IL-6 levels.

There were no significant differences in adverse events between the 2 groups, nor were any unexpected adverse outcomes identified. However, this small study cannot thoroughly or systematically evaluate the safety of canakinumab. The question of clinical efficacy clearly remains open. This question will be answered by the Phase III trial, CANTOS, which randomized >10,000 patients with hs-CRP ≥2 mg/l to treatment in a secondary prevention population; the goal is to evaluate the composite primary endpoint of nonfatal myocardial infarction, nonfatal stroke, or cardiovascular death 20, 21.

## Conclusions

In patients with T2DM and established cardiovascular disease, canakinumab reduced markers of inflammation (hs-CRP and IL-6) compared with placebo. Treatment with canakinumab also increased levels of triglycerides and total cholesterol but reduced lipoprotein(a) levels. Despite measurable effects on systemic markers of inflammation, there was no statistically significant effect on measures of vascular structure or function. Effects of canakinumab on plaque inflammation or on leukocyte function elsewhere may be undetectable with current imaging technologies. The results of this Phase II trial leave open the important question of clinical efficacy, which will be addressed by the multinational Phase III trial CANTOS.

Perspectives **COMPETENCY IN MEDICAL KNOWLEDGE:** Markers of inflammation such as C-reactive protein and serum amyloid A are related to cardiovascular prognosis in patients with atherosclerosis. Canakinumab, a monoclonal antibody that inhibits IL-1β, reduces these markers without measurable effects on arterial structure or function.

**TRANSLATIONAL OUTLOOK:** The therapeutic efficacy of canakinumab in patients with vascular disease and T2DM or glucose intolerance will be assessed in a multicenter clinical trial.

## Figures and Tables

**Central Illustration:**
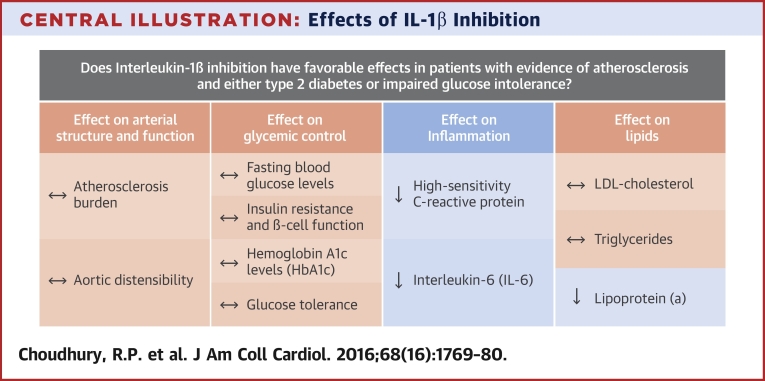
Effects of IL-1β Inhibition Interleukin (IL)-1β seems important in the pathogenesis of atherosclerosis. In this placebo-controlled trial in patients with evidence of clinical atherosclerosis and either type 2 diabetes mellitus or impaired glucose tolerance, the IL-1β inhibitor canakinumab reduced measures of inflammation but did not significantly affect measures of vascular structure or function. LDL = low-density lipoprotein.

**Table 1:** Baseline Characteristics

	Canakinumab (n = 95)	Placebo (n = 94)
Male	82 (86)	80 (85)
Age, yrs	61.7 ± 7.8	61.9 ± 6.9
Diabetes	81 (85)	81 (86)
Hypertension	81 (85)	83 (88)
Systolic blood pressure, mm Hg	128.7 ± 10.3	128.4 ± 10.0
Diastolic blood pressure, mm Hg	76.6 ± 8.1	76.9 ± 9.1
Current smoker	7 (7)	7 (7)
Peripheral vascular disease	8 (8)	8 (8)
Previous stroke	21 (22)	22 (23)
CAD	79 (83)	90 (96)
Medications		
ACE inhibitor	66 (69)	72 (77)
Beta-blocker	62 (65)	75 (80)
Statin	92 (97)	94 (100)
Insulin	29 (31)	26 (28)
Antiplatelet agent∗	90 (95)	89 (95)
BMI, kg/m^2^	30.3 ± 4.1	30.3 ± 4.0
hs-CRP, mg/l	1.77 (0.84–3.74)	1.85 (0.83–3.88)
IL-6, ng/l	2.20 (1.61–3.63)	2.24 (1.60–3.30)
Serum amyloid A, mg/l	3.8 (1.9–6.3)	3.0 (1.9–6.8)
Adiponectin, ng/ml	3,685 (2,860–5,740)	3,880 (2,620–4,950)
Lp(a), mg/dl	18.8 (4.1–37.7)	15.1 (2.0–40.7)
LDL cholesterol, mg/dl	90.5 ± 33.3	88.6 ± 36.3
HDL cholesterol, mg/dl	42.5 ± 10.4	41.8 ± 10.4
Triglycerides, mg/dl	147 ± 106	138 ± 72
Duration of diabetes, yrs		
≤1	5	7
>1 and <5	22	16
≥5	54	58
Fasting blood glucose, mg/dl	144 ± 48	146 ± 48
Insulin, pmol/l	77 (53–133)	72 (51–120)
HOMA-IR	3.7 (2.4–6.6)	3.6 (2.4–6.1)
HbA_1c_, %	7.03 ± 1.02	6.85 ± 0.93
HbA_1c_		
≤7%	50	52
>7% and <7.5%	14	20
≥7.5%	29	21

Values are n (%), mean ± SD, or median (interquartile range).

ACE = angiotensin-converting enzyme; BMI = body mass index; CAD = coronary artery disease; HbA_1c_ = glycosylated hemoglobin; HDL = high-density lipoprotein; HOMA-IR = homeostatic model assessment–insulin resistance; hs-CRP = high-sensitivity C-reactive protein; IL = interleukin; LDL = low-density lipoprotein; Lp(a) = lipoprotein(a).

∗ Aspirin, clopidogrel, prasugrel, or ticagrelor. For HbA1c, there are 2 missing values for canakinumab and 1 for the control group.

**Table 2:** Subject Disposition

	Placebo (n = 94)	Canakinumab (n = 95)	Total (N = 189)
Patients			
Completed	73 (77.7)	67 (70.5)	140 (74.1)
Discontinued∗	21 (22.3)	28 (29.5)	49 (25.9)
Main cause of discontinuation			
Adverse event(s)∗	11 (11.7)	14 (14.7)	25 (13.2)
Consent withdrawal	3 (3.2)	4 (4.2)	7 (3.7)
Lost to follow-up	1 (1.1)	2 (2.1)	3 (3.2)
Administrative	4 (4.3)	2 (2.1)	6 (3.2)
Death	0 (0)	1 (1.1)	1 (0.5)
Protocol deviation	2 (2.1)	5 (5.3)	7 (3.7)

Values are n (%).

∗ There was no significant difference between canakinumab compared with placebo for the overall rate of discontinuation from the study (risk ratio: 1.32; 95% confidence interval: 0.81 to 2.15; p = 0.26) or the rate of discontinuation due to adverse events (risk ratio: 1.26; 95% confidence interval: 0.60 to 2.63; p = 0.54).

**Table 3:** Change From Baseline in MRI Measures

	3 Months	12 Months
	No. of Patients: LSM (95% CI), p Value	No. of Patients: LSM (95% CI), p Value
Mean (R & L) carotid wall area, mm^2^		
Canakinumab	n = 63; 0.97 (–1.55 to 3.48)	n = 48; 0.59 (–2.40 to 3.59)
Placebo	n = 67; 2.13 (–0.37 to 4.64)	n = 55; 3.96 (0.94 to 6.98)
Canakinumab vs. placebo	–1.17 (–4.17 to 1.84), p = 0.44	–3.37 (–6.90 to 0.16), p = 0.06
Proximal ascending aorta wall area, mm^2^		
Canakinumab	n = 64; 2.12 (–10.61 to 14.85)	n = 50; 11.20 (–9.77 to 32.16)
Placebo	n = 69; 16.12 (2.90 to 29.34)	n = 59; 30.26 (9.57 to 50.96)
Canakinumab vs. placebo	–14.00 (–29.82 to 1.82), p = 0.08	–19.07 (–44.00 to 5.87), p = 0.13
Proximal descending aorta wall area, mm^2^		
Canakinumab	n = 75; 9.01 (–2.17 to 20.19)	n = 63; 20.92 (5.06 to 36.78)
Placebo	n = 80; 6.26 (–5.18 to 17.69)	n = 67; 25.28 (8.54 to 42.02)
Canakinumab vs. placebo	2.75 (–10.57 to 16.08), p = 0.68	–4.36 (–23.63 to 14.92), p = 0.66
Distal descending aorta wall area, mm^2^		
Canakinumab	n = 67; 3.08 (–7.08 to 13.25)	n = 56; 14.65 (0.86 to 28.45)
Placebo	n = 73; –4.41 (–14.76 to 5.95)	n = 64; 21.02 (6.67 to 35.36)
Canakinumab vs. placebo	7.49 (–4.74 to 19.72), p = 0.23	–6.36 (–23.49 to 10.77), p = 0.46
Ascending aorta distensibility, × 10^3^ mm Hg^–1^		
Canakinumab	n = 66; 0.00 (–0.26 to 0.27)	n = 51; –0.11 (–0.38 to 0.17)
Placebo	n = 61; –0.13 (–0.41 to 0.16)	n = 53; –0.13 (–0.43 to 0.17)
Canakinumab vs. placebo	0.13 (–0.20 to 0.46), p = 0.44	0.03 (–0.32 to 0.370), p = 0.87
Proximal descending aorta distensibility, × 10^3^ mm Hg^–1^		
Canakinumab	n = 70; 0.08 (–0.23 to 0.39)	n = 56; –0.19 (–0.53 to 0.16)
Placebo	n = 71; 0.10 (–0.22 to 0.41)	n = 62; –0.25 (–0.60 to 0.11)
Canakinumab vs. placebo	–0.02 (–0.39 to 0.35), p = 0.93	0.06 (–0.36 to 0.48), p = 0.78
Distal descending aorta distensibility, × 10^3^ mm Hg^–1^		
Canakinumab	n = 72; 0.04 (–0.32 to 0.40)	n = 54; –0.14 (–0.59 to 0.31)
Placebo	n = 74; 0.12 (–0.24 to 0.49)	n = 64; –0.20 (–0.66 to 0.25)
Canakinumab vs. placebo	–0.09 (–0.51 to 0.34), p = 0.69	0.06 (–0.48 to 0.61), p = 0.82

Least squared means (LSM) of outcomes (95% confidence intervals [CIs]) and number of patients in each group reported from the analysis of covariance of the change from baseline at 3 and 12 months, adjusted for baseline of outcome and including the 2-level factor type 2 diabetes mellitus or impaired glucose tolerance as a covariate. Inclusion of patients is according to the protocol. Data from an end-of-study scan of 8 patients who left the trial early were included if the time of the scan was within the allowed time limits for the 3-month scan (82 to 130 days).

L = left; MRI = magnetic resonance imaging; R = right; other abbreviations as in Table 2.

**Table 4:** Blood Measures for Lipids, Diabetes Control, and Markers of Inflammation

	3 Months	12 Months
	No. of Patients; LSM (95% CI), p Value	
LDL cholesterol, mg/dl		
Log-transformed results		
Canakinumab	n = 64; 4.425 (4.343 to 4.508)	n = 55; 4.515 (4.414 to 4.616)
Placebo	n = 75; 4.361 (4.278 to 4.444)	n = 65; 4.460 (4.357 to 4.563)
Canakinumab vs. placebo	0.065 (–0.029 to 0.158), p = 0.18	0.055 (–0.064 to 0.174), p = 0.36
Back-transformed results		
Canakinumab	83.52 (76.94 to 90.75)	91.38 (82.60 to 101.10)
Placebo	78.34 (72.10 to 85.12)	86.49 (78.03 to 95.88)
Canakinumab/placebo	1.067 (0.971 to 1.171)	1.057 (0.938 to 1.190)
Total cholesterol, mg/dl		
Log-transformed results		
Canakinumab	n = 49; 5.153 (5.098 to 5.208)	n = 40; 5.193 (5.119 to 5.267)
Placebo	n = 58; 5.040 (4.983 to 5.096)	n = 52; 5.113 (5.037 to 5.188)
Canakinumab vs. placebo	0.113 (0.048 to 0.178), p = 0.0008	0.081 (–0.009 to 0.170), p = 0.08
Back-transformed results		
Canakinumab	173.0 (163.7 to 182.7)	180.0 (167.2 to 193.8)
Placebo	154.5 (145.9 to 163.4)	166.2 (154.0 to 179.1)
Canakinumab/placebo	1.120 (1.050 to 1.195)	1.084 (0.991 to 1.185)
Triglycerides, mg/dl		
Log-transformed results		
Canakinumab	n = 73; 4.938 (4.843 to 5.033)	n = 62; 4.979 (4.865 to 5.093)
Placebo	n = 79; 4.746 (4.646 to 4.845)	n = 70; 4.795 (4.676 to 4.915)
Canakinumab vs. placebo	0.192 (0.079 to 0.306), p = 0.001	0.184 (0.045 to 0.322), p = 0.01
Back-transformed results		
Canakinumab	139.5 (126.8 to 153.4)	145.3 (129.7 to 162.9)
Placebo	115.1 (104.2 to 127.1)	120.9 (107.3 to 136.3)
Canakinumab/placebo	1.212 (1.082 to 1.358)	1.202 (1.046 to 1.380)
HDL cholesterol, mg/dl∗		
Canakinumab	n = 73; 1.779 (0.309 to 3.252)	n = 62; 0.731 (–1.017 to 2.479)
Placebo	n = 79; –0.282 (–1.817 to 1.257)	n = 70; –0.182 (–1.910 to 1.640)
Canakinumab vs. placebo	2.061 (0.302 to 3.821), p = 0.02	0.913 (–1.214 to 3.039), p = 0.40
Lp(a), mg/dl∗		
Canakinumab	n = 56; –3.325 (–5.835 to –0.816)	n = 47; –3.083 (–6.066 to –0.100)
Placebo	n = 59; 0.394 (–2.333 to 3.120)	n = 51; 1.217 (–2.059 to 4.493)
Canakinumab vs. placebo	–3.719 (–6.809 to –0.628), p = 0.02	–4.300 (–8.052 to –0.548), p = 0.025

	No. of Patients; Mean (95% CI on Log Scale)	
HbA_1c_, %		
Log-transformed results		
Canakinumab	n = 73; 1.919 (1.900 to 1.942)	n = 60; 1.930 (1.897 to 1.963)
Placebo	n = 80; 1.918 (1.894 to 1.943)	n = 69; 1.922 (1.886 to 1.958)
Canakinumab vs. placebo	0.001 (–0.025 to 0.027), p = 0.95	0.008 (–0.031 to 0.047), p = 0.68
Back-transformed results		
Canakinumab	6.814 (6.686 to 6.973)	6.890 (6.666 to 7.121)
Placebo	6.807 (6.646 to 6.980)	6.835 (6.593 to 7.085)
Canakinumab/placebo	1.001 (0.975 to 1.027)	1.008 (0.969 to 1.048)
Fasting blood glucose, mg/dl		
Log-transformed results		
Canakinumab	n = 71; 4.928 (4.868 to 4.989)	n = 58; 4.922 (4.845 to 4.999)
Placebo	n = 78; 4.849 (4.786 to 4.912)	n = 68; 4.853 (4.773 to 4.934)
Canakinumab vs. placebo	0.079 (0.013 to 0.145), p = 0.02	0.068 (–0.017 to 0.154), p = 0.12
Back-transformed results		
Canakinumab	138 (130 to 147)	137 (127 to 148)
Placebo	128 (120 to 136)	128 (118 to 139)
Canakinumab/placebo	1.082 (1.013 to 1.156)	1.070 (0.983 to 1.166)
HOMA-IR		
Log-transformed results		
Canakinumab	n = 67; 1.443 (1.282 to 1.603)	n = 56; 1.324 (1.133 to 1.514)
Placebo	n = 76; 1.358 (1.190 to 1.527)	n = 68; 1.278 (1.082 to 1.473)
Canakinumab vs. placebo	0.084 (–0.094 to 0.262), p = 0.35	0.046 (–0.170 to 0.262), p = 0.67
Back-transformed results		
Canakinumab	4.233 (3.604 to 4.968)	3.758 (3.105 to 4.545)
Placebo	3.888 (3.287 to 4.604)	3.589 (2.951 to 4.362)
Canakinumab/placebo	1.088 (0.910 to 1.300)	1.047 (0.844 to 1.300)
HOMA-B		
Log-transformed results		
Canakinumab	n = 66; 4.066 (3.886 to 4.247)	n = 56; 3.969 (3.773 to 4.165)
Placebo	n = 76; 4.224 (4.033 to 4.415)	n = 68; 4.133 (3.929 to 4.337)
Canakinumab vs. placebo	–0.158 (–0.361 to 0.046), p = 0.13	–0.164 (–0.389 to 0.062), p = 0.15
Back-transformed results		
Canakinumab	58.32 (48.72 to 69.90)	52.93 (43.51 to 64.39)
Placebo	68.31 (56.43 to 82.68)	62.36 (50.86 to 76.48)
Canakinumab/placebo	0.854 (0.697 to 1.047)	0.849 (0.678 to 1.064)
C-reactive protein, mg/l		
Log-transformed results		
Canakinumab	n = 74; –0.278 (–0.500 to –0.056)	n = 61; –0.170 (–0.419 to 0.078)
Placebo	n = 79; 0.287 (0.051 to 0.523)	n = 69; 0.409 (0.145 to 0.673)
Canakinumab vs. placebo	–0.565 (–0.829 to –0.301), p < 0.0001	–0.579 (–0.881 to –0.277), p = 0.0002
Back-transformed results		
Canakinumab	0.757 (0.607 to 0.946)	0.848 (0.658 to 1.081)
Placebo	1.332 (1.052 to 1.687)	1.505 (1.156 to 1.9690)
Canakinumab/placebo	0.568 (0.436 to 0.740)	0.561 (0.414 to 0.758)
IL-6, pg/ml		
Log-transformed results		
Canakinumab	n = 48; 0.367 (0.205 to 0.529)	n = 13; 0.744 (0.313 to 1.745)
Placebo	n = 48; 0.913 (0.742 to 1.081)	n = 14; 1.147 (0.729 to 1.565)
Canakinumab vs. placebo	–0.544 (–0.728 to –0.361), p < 0.0001	–0.403 (–0.888 to 0.081), p = 0.10
Back-transformed results		
Canakinumab	1.443 (1.228 to 1.697)	2.104 (1.368 to 5.726)
Placebo	2.492 (2.100 to 2.948)	3.149 (2.073 to 4.783)
Canakinumab/placebo	0.580 (0.483 to 0.697)	0.668 (0.411 to 1.084)
Serum amyloid A, mg/l		
Log-transformed results		
Canakinumab	n = 67; 0.963 (0.760 to 1.166)	n = 56; 1.000 (0.795 to 1.205)
Placebo	n = 76; 1.129 (0.922 to 1.336)	n = 69; 1.243 (1.032 to 1.453)
Canakinumab vs. placebo	–0.166 (–0.408 to 0.077), p = 0.18	–0.243 (–0.493 to 0.008), p = 0.06
Back-transformed results		
Canakinumab	2.620 (2.138 to 3.209)	2.718 (2.214 to 3.337)
Placebo	3.093 (2.514 to 3.804)	3.466 (2.807 to 4.276)
Canakinumab/placebo	0.847 (0.665 to 1.080)	0.784 (0.611 to 1.008)
Adiponectin, ng/ml		
Log-transformed results		
Canakinumab	n = 36; 8.265 (8.208 to 8.323)	
Placebo	n = 36; 8.284 (8.227 to 8.342)	
Canakinumab vs. placebo	–0.019 (–0.084 to 0.046), p = 0.57	
Back-transformed results		
Canakinumab	3,885 (3,670 to 4,117)	
Placebo	3,960 (3,741 to 4,196)	
Canakinumab/placebo	0.981 (0.919 to 1.047)	

Abbreviations as in Tables 1 and 3.

∗ Change from baseline, not log-transformed. LSMs of outcomes (95% CIs) with number of patients reported from the analysis of covariance of the log-transformed outcome at 3 and 12 months, adjusted for log-transformed baseline of outcome and including the 2-level factor type 2 diabetes mellitus or impaired glucose tolerance as a covariate. Inclusion of patients is according to the protocol. The treatment effect is reported as the difference between the canakinumab arm and the placebo arm and after back-transforming the treatment effect as the ratio of the levels in the canakinumab arm to the placebo arm. The LSMs are back-transformed (geometric means).

**Table 5:** MACE

	MACE	Placebo	Canakinumab	p Value
Group				
All				
N		94	95	
	Yes	7 (7)	10 (11)	0.612
	No	87 (93)	85 (89)	
Type 2 diabetes mellitus				
N		81	81	
	Yes	6 (7)	9 (11)	0.589
	No	75 (93)	72 (89)	
Impaired glucose tolerance				
N		13	14	
	Yes	1 (8)	1 (7)	1.000
	No	12 (92)	13 (93)	

Values are n (%) unless otherwise indicated.

MACE = major adverse cardiac events.
