# Supplementary material for: Arterial Effects of Canakinumab in Patients With Atherosclerosis and Type 2 Diabetes or Glucose Intolerance
Source: J Am Coll Cardiol. 2016 Oct 18;68(16):1769–80. doi: 10.1016/j.jacc.2016.07.768 (PMC5064025; doi:10.1016/j.jacc.2016.07.768)
Supplement: Online Data [file mmc1.docx]

**ONLINE APPENDIX**

**IMAGING PROTOCOLS AND ANALYSIS**

For aortic stiffness measures including strain and distensibility, 2-axial, electrocardiogram (ECG)-gated steady-state-free precession image sets were acquired during breath-hold to cover 3 distinct aortic regions, including the proximal ascending aorta (PAA), the proximal descending aorta (PDA), and distal descending aorta (DDA). The first image set was obtained at the level of the right pulmonary artery, covering the PAA and PDA, while the second set to cover the DDA was obtained just above the diaphragm with 11 cm separating the center slice position of the proximal and distal descending aortic locations. To assess plaque burden in the same aortic regions, proton density-weighted (PDW) ECG-gated double inversion recovery black blood fast spin echocardiographic images were obtained during breath-hold using 2 sets of 3 slices placed perpendicular to the vessel and approximately axial with slice thickness of 5 mm and interslice gap of 5 mm. The 2 slice groups utilized the same aortic reference points as for the cine TrueFISP (Siemens) acquisitions for the aortic stiffness measures.

For assessment of the plaque burden within the carotid arteries, axial ECG-gated multi-contrast black blood images were obtained with T1 weighting (T1W), PDW, and T2 weighting (T2W). The right carotid bifurcation was used as the internal landmark for a bilateral acquisition. A range of 6 to 13 slices was acquired for each of the contrast weightings. For T1W images, the repetition time was determined by 1 R-R interval and for T2W and PDW 2 R-R intervals. Both the carotid and aortic magnetic resonance images were acquired in the same scan session and required 60 to 75 min for total acquisition time including the MRI coil changes.

Imaging data were collated by Bioclinica (Leiden, the Netherlands) and distributed to the image analysis core laboratories. Vessel wall area (VWA) was analyzed at a central core laboratory by an experienced observer blinded to the type of exam (baseline vs. follow-up) and to treatment allocation. The vessel wall boundaries (i.e., the outer and inner wall contours) were manually traced to derive vessel dimensions and morphologies, as in previous studies (1). Based on these contours, software (VesselMASS, Leiden, the Netherlands) automatically calculated lumen area (LA) and total vessel area (TVA). The VWA was computed by subtracting the LA from the TVA. To obtain a single carotid value, measurements were averaged for the left and right common carotid arteries. As all imaging time points were analyzed independently, no coregistration across time points was performed, and only the common carotid in the region of the bifurcation was analyzed.

Semi-automated analysis of cine MRI was performed using software developed in Matlab (Mathworks, Natick, Massachusetts) and based on validated algorithms at a central core laboratory (2). Arterial function was characterized by distensibility of the PAA, PDA, and DDA. Maximum (MAXarea) and minimum (MINarea) LA measurements were extracted from ECG-gated series of bright-blood cross-sectional images acquired throughout the cardiac cycle. Arterial strain was given by (MAXarea-MINarea)/MINarea*.* Distensibility was calculated as strain divided by central pulse pressure measured by SphygmoCor (AtCor, Sydney, Australia).

References

(1) Fayad ZA, Mani V, Woodward M, et al. Safety and efficacy of dalcetrapib on atherosclerotic disease using novel non-invasive multimodality imaging (dal-PLAQUE): a randomised clinical trial. Lancet 2011;378:1547-59.

(2) Jackson CE, Shirodaria CC, Lee JM, et al. Reproducibility and accuracy of automated measurement for dynamic arterial lumen area by cardiovascular magnetic resonance. Int J Cardiovasc Imaging. 2009;25:797-808.

**Supplementary Table 1**

**Overall adverse events - n (%) of subjects**

|  | **Placebo N=94**  **n (%)** | **Canakinumab N=95**  **n (%)** | **Total N=189**  **n (%)** |
| --- | --- | --- | --- |
| Patients with AE(s) | 80 (85.1%) | 77 (81.1%) | 157 (83.1%) |
| **Preferred term** |  |  |  |
| Nasopharyngitis | 18 (19.1%) | 12 (12.6%) | 30 (15.9%) |
| Upper respiratory tract infection | 10 (10.6%) | 9 (9.5%) | 19 (10.1%) |
| Dizziness | 5 (5.3%) | 6 (6.3%) | 11 (5.8%) |
| Diarrhea | 6 (6.4%) | 5 (5.3%) | 11 (5.8%) |
| Blood creatine phosphokinase increased | 3 (3.2%) | 5 (5.3%) | 8 (4.2%) |
| Cough | 2 (2.1%) | 5 (5.3%) | 7 (3.7%) |
| Urinary tract infection | 1 (1.1%) | 5 (5.3%) | 6 (3.2%) |
| Non-cardiac chest pain | 8 (8.5%) | 4 (4.2%) | 12 (6.3%) |
| Musculoskeletal pain | 7 (7.4%) | 4 (4.2%) | 11 (5.8%) |
| Nausea | 7 (7.4%) | 4 (4.2%) | 11 (5.8%) |
| Back pain | 6 (6.4%) | 4 (4.2%) | 10 (5.3%) |
| Headache | 5 (5.3%) | 4 (4.2%) | 9 (4.8%) |
| Influenza like illness | 5 (5.3%) | 4 (4.2%) | 9 (4.8%) |
| Vertigo | 0 (0.0%) | 4 (4.2%) | 4 (2.1%) |
| Renal failure | 6 (6.4%) | 3 (3.2%) | 9 (4.8%) |
| Hypertension | 5 (5.3%) | 3 (3.2%) | 8 (4.2%) |
| C-reactive protein increased | 4 (4.3%) | 3 (3.2%) | 7 (3.7%) |
| Myalgia | 3 (3.2%) | 3 (3.2%) | 6 (3.2%) |
| Diabetes mellitus inadequate control | 2 (2.1%) | 3 (3.2%) | 5 (2.6%) |
| Hyperglycaemia | 2 (2.1%) | 3 (3.2%) | 5 (2.6%) |
| Influenza | 2 (2.1%) | 3 (3.2%) | 5 (2.6%) |
| Lipase increased | 2 (2.1%) | 3 (3.2%) | 5 (2.6%) |
| Haematuria | 1 (1.1%) | 3 (3.2%) | 4 (2.1%) |
| Laceration | 0 (0.0%) | 3 (3.2%) | 3 (1.6%) |
| Neck pain | 0 (0.0%) | 3 (3.2%) | 3 (1.6%) |
| Fatigue | 8 (8.5%) | 2 (2.1%) | 10 (5.3%) |
| Pruritus | 6 (6.4%) | 2 (2.1%) | 8 (4.2%) |
| Asthenia | 5 (5.3%) | 2 (2.1%) | 7 (3.7%) |
| Angina pectoris | 4 (4.3%) | 2 (2.1%) | 6 (3.2%) |
| Gastroenteritis | 4 (4.3%) | 2 (2.1%) | 6 (3.2%) |
| Arthralgia | 3 (3.2%) | 2 (2.1%) | 5 (2.6%) |
| Bronchitis | 3 (3.2%) | 2 (2.1%) | 5 (2.6%) |
| Hyperamylasaemia | 3 (3.2%) | 2 (2.1%) | 5 (2.6%) |
| Hyperlipasaemia | 3 (3.2%) | 2 (2.1%) | 5 (2.6%) |
| Hypoglycaemia | 3 (3.2%) | 2 (2.1%) | 5 (2.6%) |
| Osteoarthritis | 3 (3.2%) | 2 (2.1%) | 5 (2.6%) |
| Anemia | 2 (2.1%) | 2 (2.1%) | 4 (2.1%) |
| Carotid artery stenosis | 2 (2.1%) | 2 (2.1%) | 4 (2.1%) |
| Seasonal allergy | 2 (2.1%) | 2 (2.1%) | 4 (2.1%) |
| Acute myocardial infarction | 1 (1.1%) | 2 (2.1%) | 3 (1.6%) |
| Gingivitis | 1 (1.1%) | 2 (2.1%) | 3 (1.6%) |
| Leukocytosis | 1 (1.1%) | 2 (2.1%) | 3 (1.6%) |
| Toothache | 1 (1.1%) | 2 (2.1%) | 3 (1.6%) |
| Urinary retention | 1 (1.1%) | 2 (2.1%) | 3 (1.6%) |
| Actinic keratosis | 0 (0.0%) | 2 (2.1%) | 2 (1.1%) |
| Benign prostatic hyperplasia | 0 (0.0%) | 2 (2.1%) | 2 (1.1%) |
| Constipation | 0 (0.0%) | 2 (2.1%) | 2 (1.1%) |
| Dermatitis contact | 0 (0.0%) | 2 (2.1%) | 2 (1.1%) |
| Joint injury | 0 (0.0%) | 2 (2.1%) | 2 (1.1%) |
| Nasal congestion | 0 (0.0%) | 2 (2.1%) | 2 (1.1%) |
| Oral herpes | 0 (0.0%) | 2 (2.1%) | 2 (1.1%) |
| Pleural effusion | 0 (0.0%) | 2 (2.1%) | 2 (1.1%) |
| Renal colic | 0 (0.0%) | 2 (2.1%) | 2 (1.1%) |
| Tachycardia | 0 (0.0%) | 2 (2.1%) | 2 (1.1%) |
| Transient ischaemic attack | 0 (0.0%) | 2 (2.1%) | 2 (1.1%) |
| Hypertriglyceridaemia | 5 (5.3%) | 1 (1.1%) | 6 (3.2%) |
| Abdominal pain | 4 (4.3%) | 1 (1.1%) | 5 (2.6%) |
| Contusion | 4 (4.3%) | 1 (1.1%) | 5 (2.6%) |
| Gastrooesophageal reflux disease | 4 (4.3%) | 1 (1.1%) | 5 (2.6%) |
| Dyspepsia | 3 (3.2%) | 1 (1.1%) | 4 (2.1%) |
| Dyspnoea | 3 (3.2%) | 1 (1.1%) | 4 (2.1%) |
| Muscle spasms | 3 (3.2%) | 1 (1.1%) | 4 (2.1%) |
| Oropharyngeal pain | 3 (3.2%) | 1 (1.1%) | 4 (2.1%) |
| Pain in extremity | 3 (3.2%) | 1 (1.1%) | 4 (2.1%) |
| Alanine aminotransferase increased | 2 (2.1%) | 1 (1.1%) | 3 (1.6%) |
| Atrial fibrillation | 2 (2.1%) | 1 (1.1%) | 3 (1.6%) |
| Cardiac failure | 2 (2.1%) | 1 (1.1%) | 3 (1.6%) |
| Gamma-glutamyltransferase increased | 2 (2.1%) | 1 (1.1%) | 3 (1.6%) |
| Gastrointestinal infection | 2 (2.1%) | 1 (1.1%) | 3 (1.6%) |
| Haemorrhoids | 2 (2.1%) | 1 (1.1%) | 3 (1.6%) |
| Hypokalaemia | 2 (2.1%) | 1 (1.1%) | 3 (1.6%) |
| Iron deficiency anaemia | 2 (2.1%) | 1 (1.1%) | 3 (1.6%) |
| Rhinorrhoea | 2 (2.1%) | 1 (1.1%) | 3 (1.6%) |
| Sinusitis | 2 (2.1%) | 1 (1.1%) | 3 (1.6%) |
| Tinnitus | 2 (2.1%) | 1 (1.1%) | 3 (1.6%) |
| Aspartate aminotransferase increased | 1 (1.1%) | 1 (1.1%) | 2 (1.1%) |
| Blood bilirubin increased | 1 (1.1%) | 1 (1.1%) | 2 (1.1%) |
| Blood glucose increased | 1 (1.1%) | 1 (1.1%) | 2 (1.1%) |
| Blood triglycerides increased | 1 (1.1%) | 1 (1.1%) | 2 (1.1%) |
| Cellulitis | 1 (1.1%) | 1 (1.1%) | 2 (1.1%) |
| Diverticulum intestinal | 1 (1.1%) | 1 (1.1%) | 2 (1.1%) |
| Dyspnoea exertional | 1 (1.1%) | 1 (1.1%) | 2 (1.1%) |
| Eczema | 1 (1.1%) | 1 (1.1%) | 2 (1.1%) |
| Epistaxis | 1 (1.1%) | 1 (1.1%) | 2 (1.1%) |
| Fall | 1 (1.1%) | 1 (1.1%) | 2 (1.1%) |
| Hypercalcaemia | 1 (1.1%) | 1 (1.1%) | 2 (1.1%) |
| Leukopenia | 1 (1.1%) | 1 (1.1%) | 2 (1.1%) |
| Paraesthesia | 1 (1.1%) | 1 (1.1%) | 2 (1.1%) |
| Periodontitis | 1 (1.1%) | 1 (1.1%) | 2 (1.1%) |
| Pneumonia | 1 (1.1%) | 1 (1.1%) | 2 (1.1%) |
| Prostatitis | 1 (1.1%) | 1 (1.1%) | 2 (1.1%) |
| Sinus congestion | 1 (1.1%) | 1 (1.1%) | 2 (1.1%) |
| Sinus tachycardia | 1 (1.1%) | 1 (1.1%) | 2 (1.1%) |
| Thrombocytopenia | 1 (1.1%) | 1 (1.1%) | 2 (1.1%) |
| Urticaria | 1 (1.1%) | 1 (1.1%) | 2 (1.1%) |
| Adrenal neoplasm | 0 (0.0%) | 1 (1.1%) | 1 (0.5%) |
| Allergic transfusion reaction | 0 (0.0%) | 1 (1.1%) | 1 (0.5%) |
| Angina unstable | 0 (0.0%) | 1 (1.1%) | 1 (0.5%) |
| Animal bite | 0 (0.0%) | 1 (1.1%) | 1 (0.5%) |
| Anxiety | 0 (0.0%) | 1 (1.1%) | 1 (0.5%) |
| Basal cell carcinoma | 0 (0.0%) | 1 (1.1%) | 1 (0.5%) |
| Bladder obstruction | 0 (0.0%) | 1 (1.1%) | 1 (0.5%) |
| Blood glucose fluctuation | 0 (0.0%) | 1 (1.1%) | 1 (0.5%) |
| Calculus bladder | 0 (0.0%) | 1 (1.1%) | 1 (0.5%) |
| Calculus ureteric | 0 (0.0%) | 1 (1.1%) | 1 (0.5%) |
| Cardiac failure congestive | 0 (0.0%) | 1 (1.1%) | 1 (0.5%) |
| Carotid artery occlusion | 0 (0.0%) | 1 (1.1%) | 1 (0.5%) |
| Cataract cortical | 0 (0.0%) | 1 (1.1%) | 1 (0.5%) |
| Chest pain | 0 (0.0%) | 1 (1.1%) | 1 (0.5%) |
| Claustrophobia | 0 (0.0%) | 1 (1.1%) | 1 (0.5%) |
| Conjunctivitis | 0 (0.0%) | 1 (1.1%) | 1 (0.5%) |
| Deep vein thrombosis | 0 (0.0%) | 1 (1.1%) | 1 (0.5%) |
| Depressed mood | 0 (0.0%) | 1 (1.1%) | 1 (0.5%) |
| Device related infection | 0 (0.0%) | 1 (1.1%) | 1 (0.5%) |
| Diabetic foot | 0 (0.0%) | 1 (1.1%) | 1 (0.5%) |
| Eosinophilia | 0 (0.0%) | 1 (1.1%) | 1 (0.5%) |
| Epididymitis | 0 (0.0%) | 1 (1.1%) | 1 (0.5%) |
| Epiglottitis | 0 (0.0%) | 1 (1.1%) | 1 (0.5%) |
| Erectile dysfunction | 0 (0.0%) | 1 (1.1%) | 1 (0.5%) |
| Exostosis | 0 (0.0%) | 1 (1.1%) | 1 (0.5%) |
| Eye discharge | 0 (0.0%) | 1 (1.1%) | 1 (0.5%) |
| Flank pain | 0 (0.0%) | 1 (1.1%) | 1 (0.5%) |
| Furuncle | 0 (0.0%) | 1 (1.1%) | 1 (0.5%) |
| Gingival infection | 0 (0.0%) | 1 (1.1%) | 1 (0.5%) |
| Glycosuria | 0 (0.0%) | 1 (1.1%) | 1 (0.5%) |
| Gouty tophus | 0 (0.0%) | 1 (1.1%) | 1 (0.5%) |
| Hepatic cancer | 0 (0.0%) | 1 (1.1%) | 1 (0.5%) |
| Hepatic enzyme increased | 0 (0.0%) | 1 (1.1%) | 1 (0.5%) |
| Hot flush | 0 (0.0%) | 1 (1.1%) | 1 (0.5%) |
| Hydrocephalus | 0 (0.0%) | 1 (1.1%) | 1 (0.5%) |
| Hyperlipidaemia | 0 (0.0%) | 1 (1.1%) | 1 (0.5%) |
| Hyperthyroidism | 0 (0.0%) | 1 (1.1%) | 1 (0.5%) |
| Hypomagnesaemia | 0 (0.0%) | 1 (1.1%) | 1 (0.5%) |
| Hypothyroidism | 0 (0.0%) | 1 (1.1%) | 1 (0.5%) |
| Infected dermal cyst | 0 (0.0%) | 1 (1.1%) | 1 (0.5%) |
| Inguinal hernia | 0 (0.0%) | 1 (1.1%) | 1 (0.5%) |
| Intraventricular hemorrhage | 0 (0.0%) | 1 (1.1%) | 1 (0.5%) |
| Joint effusion | 0 (0.0%) | 1 (1.1%) | 1 (0.5%) |
| Kidney infection | 0 (0.0%) | 1 (1.1%) | 1 (0.5%) |
| Lethargy | 0 (0.0%) | 1 (1.1%) | 1 (0.5%) |
| Lip dry | 0 (0.0%) | 1 (1.1%) | 1 (0.5%) |
| Lipoma | 0 (0.0%) | 1 (1.1%) | 1 (0.5%) |
| Localized infection | 0 (0.0%) | 1 (1.1%) | 1 (0.5%) |
| Low density lipoprotein decreased | 0 (0.0%) | 1 (1.1%) | 1 (0.5%) |
| Lymph node pain | 0 (0.0%) | 1 (1.1%) | 1 (0.5%) |
| Malaise | 0 (0.0%) | 1 (1.1%) | 1 (0.5%) |
| Malignant melanoma | 0 (0.0%) | 1 (1.1%) | 1 (0.5%) |
| Melanocytic nevus | 0 (0.0%) | 1 (1.1%) | 1 (0.5%) |
| Muscle contracture | 0 (0.0%) | 1 (1.1%) | 1 (0.5%) |
| Musculoskeletal chest pain | 0 (0.0%) | 1 (1.1%) | 1 (0.5%) |
| Myelodysplastic syndrome | 0 (0.0%) | 1 (1.1%) | 1 (0.5%) |
| Myocardial ischemia | 0 (0.0%) | 1 (1.1%) | 1 (0.5%) |
| Nephrolithiasis | 0 (0.0%) | 1 (1.1%) | 1 (0.5%) |
| Ophthalmic herpes simplex | 0 (0.0%) | 1 (1.1%) | 1 (0.5%) |
| Oral candidiasis | 0 (0.0%) | 1 (1.1%) | 1 (0.5%) |
| Paronychia | 0 (0.0%) | 1 (1.1%) | 1 (0.5%) |
| Peripheral artery thrombosis | 0 (0.0%) | 1 (1.1%) | 1 (0.5%) |
| Peripheral vascular disorder | 0 (0.0%) | 1 (1.1%) | 1 (0.5%) |
| Pollakiuria | 0 (0.0%) | 1 (1.1%) | 1 (0.5%) |
| Prothrombin time prolonged | 0 (0.0%) | 1 (1.1%) | 1 (0.5%) |
| Psoriasis | 0 (0.0%) | 1 (1.1%) | 1 (0.5%) |
| Pulpitis dental | 0 (0.0%) | 1 (1.1%) | 1 (0.5%) |
| Pyrexia | 0 (0.0%) | 1 (1.1%) | 1 (0.5%) |
| Rectal hemorrhage | 0 (0.0%) | 1 (1.1%) | 1 (0.5%) |
| Respiratory tract congestion | 0 (0.0%) | 1 (1.1%) | 1 (0.5%) |
| Salivary gland pain | 0 (0.0%) | 1 (1.1%) | 1 (0.5%) |
| Sarcoidosis | 0 (0.0%) | 1 (1.1%) | 1 (0.5%) |
| Sciatica | 0 (0.0%) | 1 (1.1%) | 1 (0.5%) |
| Serum amyloid a protein increased | 0 (0.0%) | 1 (1.1%) | 1 (0.5%) |
| Sinus bradycardia | 0 (0.0%) | 1 (1.1%) | 1 (0.5%) |
| Squamous cell carcinoma of skin | 0 (0.0%) | 1 (1.1%) | 1 (0.5%) |
| Subdural hematoma | 0 (0.0%) | 1 (1.1%) | 1 (0.5%) |
| Sudden hearing loss | 0 (0.0%) | 1 (1.1%) | 1 (0.5%) |
| Syncope | 0 (0.0%) | 1 (1.1%) | 1 (0.5%) |
| Systemic inflammatory response syndrome | 0 (0.0%) | 1 (1.1%) | 1 (0.5%) |
| Tooth abscess | 0 (0.0%) | 1 (1.1%) | 1 (0.5%) |
| Type 2 diabetes mellitus | 0 (0.0%) | 1 (1.1%) | 1 (0.5%) |
| Weight decreased | 0 (0.0%) | 1 (1.1%) | 1 (0.5%) |
| Vomiting | 5 (5.3%) | 0 (0.0%) | 5 (2.6%) |
| Decreased appetite | 3 (3.2%) | 0 (0.0%) | 3 (1.6%) |
| Rash | 3 (3.2%) | 0 (0.0%) | 3 (1.6%) |
| Coronary artery disease | 2 (2.1%) | 0 (0.0%) | 2 (1.1%) |
| Cystitis | 2 (2.1%) | 0 (0.0%) | 2 (1.1%) |
| Dermatitis allergic | 2 (2.1%) | 0 (0.0%) | 2 (1.1%) |
| Dry skin | 2 (2.1%) | 0 (0.0%) | 2 (1.1%) |
| Dysuria | 2 (2.1%) | 0 (0.0%) | 2 (1.1%) |
| Herpes zoster | 2 (2.1%) | 0 (0.0%) | 2 (1.1%) |
| Hiatus hernia | 2 (2.1%) | 0 (0.0%) | 2 (1.1%) |
| Hypotension | 2 (2.1%) | 0 (0.0%) | 2 (1.1%) |
| Edema peripheral | 2 (2.1%) | 0 (0.0%) | 2 (1.1%) |
| Pulmonary edema | 2 (2.1%) | 0 (0.0%) | 2 (1.1%) |
| Rhinitis | 2 (2.1%) | 0 (0.0%) | 2 (1.1%) |
| Abdominal distension | 1 (1.1%) | 0 (0.0%) | 1 (0.5%) |
| Abscess jaw | 1 (1.1%) | 0 (0.0%) | 1 (0.5%) |
| Acute coronary syndrome | 1 (1.1%) | 0 (0.0%) | 1 (0.5%) |
| Amylase increased | 1 (1.1%) | 0 (0.0%) | 1 (0.5%) |
| Arthritis | 1 (1.1%) | 0 (0.0%) | 1 (0.5%) |
| Arthropod bite | 1 (1.1%) | 0 (0.0%) | 1 (0.5%) |
| Bacteriuria | 1 (1.1%) | 0 (0.0%) | 1 (0.5%) |
| Barrett's esophagus | 1 (1.1%) | 0 (0.0%) | 1 (0.5%) |
| Blood alkaline phosphatase increased | 1 (1.1%) | 0 (0.0%) | 1 (0.5%) |
| Blood lactate dehydrogenase increased | 1 (1.1%) | 0 (0.0%) | 1 (0.5%) |
| Blood potassium increased | 1 (1.1%) | 0 (0.0%) | 1 (0.5%) |
| Blood testosterone decreased | 1 (1.1%) | 0 (0.0%) | 1 (0.5%) |
| Blood uric acid increased | 1 (1.1%) | 0 (0.0%) | 1 (0.5%) |
| Bradycardia | 1 (1.1%) | 0 (0.0%) | 1 (0.5%) |
| Breast cancer | 1 (1.1%) | 0 (0.0%) | 1 (0.5%) |
| Carotid bruit | 1 (1.1%) | 0 (0.0%) | 1 (0.5%) |
| Cataract | 1 (1.1%) | 0 (0.0%) | 1 (0.5%) |
| Cerumen impaction | 1 (1.1%) | 0 (0.0%) | 1 (0.5%) |
| Cholecystitis acute | 1 (1.1%) | 0 (0.0%) | 1 (0.5%) |
| Convulsion | 1 (1.1%) | 0 (0.0%) | 1 (0.5%) |
| Coronary artery restenosis | 1 (1.1%) | 0 (0.0%) | 1 (0.5%) |
| Deafness | 1 (1.1%) | 0 (0.0%) | 1 (0.5%) |
| Dental necrosis | 1 (1.1%) | 0 (0.0%) | 1 (0.5%) |
| Dizziness postural | 1 (1.1%) | 0 (0.0%) | 1 (0.5%) |
| Ear infection | 1 (1.1%) | 0 (0.0%) | 1 (0.5%) |
| Eczema nummular | 1 (1.1%) | 0 (0.0%) | 1 (0.5%) |
| Electrocardiogram pr prolongation | 1 (1.1%) | 0 (0.0%) | 1 (0.5%) |
| Electrocardiogram st-t change | 1 (1.1%) | 0 (0.0%) | 1 (0.5%) |
| Eye pain | 1 (1.1%) | 0 (0.0%) | 1 (0.5%) |
| Femur fracture | 1 (1.1%) | 0 (0.0%) | 1 (0.5%) |
| Flatulence | 1 (1.1%) | 0 (0.0%) | 1 (0.5%) |
| Gait disturbance | 1 (1.1%) | 0 (0.0%) | 1 (0.5%) |
| Gastritis erosive | 1 (1.1%) | 0 (0.0%) | 1 (0.5%) |
| Gastrointestinal mucosal disorder | 1 (1.1%) | 0 (0.0%) | 1 (0.5%) |
| Gout | 1 (1.1%) | 0 (0.0%) | 1 (0.5%) |
| Hand fracture | 1 (1.1%) | 0 (0.0%) | 1 (0.5%) |
| Helicobacter test positive | 1 (1.1%) | 0 (0.0%) | 1 (0.5%) |
| Hyperhidrosis | 1 (1.1%) | 0 (0.0%) | 1 (0.5%) |
| Hyperuricaemia | 1 (1.1%) | 0 (0.0%) | 1 (0.5%) |
| Hypoesthesia | 1 (1.1%) | 0 (0.0%) | 1 (0.5%) |
| Inguinal hernia, obstructive | 1 (1.1%) | 0 (0.0%) | 1 (0.5%) |
| Injection site hematoma | 1 (1.1%) | 0 (0.0%) | 1 (0.5%) |
| Insomnia | 1 (1.1%) | 0 (0.0%) | 1 (0.5%) |
| Intervertebral disc protrusion | 1 (1.1%) | 0 (0.0%) | 1 (0.5%) |
| Joint dislocation | 1 (1.1%) | 0 (0.0%) | 1 (0.5%) |
| Large intestine polyp | 1 (1.1%) | 0 (0.0%) | 1 (0.5%) |
| Left ventricular dysfunction | 1 (1.1%) | 0 (0.0%) | 1 (0.5%) |
| Lichen planus | 1 (1.1%) | 0 (0.0%) | 1 (0.5%) |
| Ligament rupture | 1 (1.1%) | 0 (0.0%) | 1 (0.5%) |
| Ligament sprain | 1 (1.1%) | 0 (0.0%) | 1 (0.5%) |
| Local swelling | 1 (1.1%) | 0 (0.0%) | 1 (0.5%) |
| Lower respiratory tract infection | 1 (1.1%) | 0 (0.0%) | 1 (0.5%) |
| Lumbar spinal stenosis | 1 (1.1%) | 0 (0.0%) | 1 (0.5%) |
| Macular degeneration | 1 (1.1%) | 0 (0.0%) | 1 (0.5%) |
| Magnesium deficiency | 1 (1.1%) | 0 (0.0%) | 1 (0.5%) |
| Micturition urgency | 1 (1.1%) | 0 (0.0%) | 1 (0.5%) |
| Musculoskeletal discomfort | 1 (1.1%) | 0 (0.0%) | 1 (0.5%) |
| Myopathy | 1 (1.1%) | 0 (0.0%) | 1 (0.5%) |
| Orthostatic hypotension | 1 (1.1%) | 0 (0.0%) | 1 (0.5%) |
| Otitis media | 1 (1.1%) | 0 (0.0%) | 1 (0.5%) |
| Palpitations | 1 (1.1%) | 0 (0.0%) | 1 (0.5%) |
| Paranasal sinus hypersecretion | 1 (1.1%) | 0 (0.0%) | 1 (0.5%) |
| Pharyngitis | 1 (1.1%) | 0 (0.0%) | 1 (0.5%) |
| Post herpetic neuralgia | 1 (1.1%) | 0 (0.0%) | 1 (0.5%) |
| Rales | 1 (1.1%) | 0 (0.0%) | 1 (0.5%) |
| Rash maculo-papular | 1 (1.1%) | 0 (0.0%) | 1 (0.5%) |
| Rebound effect | 1 (1.1%) | 0 (0.0%) | 1 (0.5%) |
| Rectal polyp | 1 (1.1%) | 0 (0.0%) | 1 (0.5%) |
| Renal failure acute | 1 (1.1%) | 0 (0.0%) | 1 (0.5%) |
| Road traffic accident | 1 (1.1%) | 0 (0.0%) | 1 (0.5%) |
| Sacroiliitis | 1 (1.1%) | 0 (0.0%) | 1 (0.5%) |
| Sleep disorder | 1 (1.1%) | 0 (0.0%) | 1 (0.5%) |
| Somnolence | 1 (1.1%) | 0 (0.0%) | 1 (0.5%) |
| Subcutaneous abscess | 1 (1.1%) | 0 (0.0%) | 1 (0.5%) |
| Thrombocytosis | 1 (1.1%) | 0 (0.0%) | 1 (0.5%) |
| Tooth fracture | 1 (1.1%) | 0 (0.0%) | 1 (0.5%) |
| Tympanic membrane perforation | 1 (1.1%) | 0 (0.0%) | 1 (0.5%) |
| Urine ketone body present | 1 (1.1%) | 0 (0.0%) | 1 (0.5%) |
| Vasodilatation | 1 (1.1%) | 0 (0.0%) | 1 (0.5%) |
| Ventricular arrhythmia | 1 (1.1%) | 0 (0.0%) | 1 (0.5%) |
| Vessel puncture site pain | 1 (1.1%) | 0 (0.0%) | 1 (0.5%) |
| Vitamin b complex deficiency | 1 (1.1%) | 0 (0.0%) | 1 (0.5%) |
| Vitamin b12 deficiency | 1 (1.1%) | 0 (0.0%) | 1 (0.5%) |
| Vitamin d deficiency | 1 (1.1%) | 0 (0.0%) | 1 (0.5%) |
| Weight increased | 1 (1.1%) | 0 (0.0%) | 1 (0.5%) |
